# Arthritis in patients with very early systemic sclerosis: a comprehensive clinical and prognostic analysis

**DOI:** 10.1093/rheumatology/keae247

**Published:** 2024-05-09

**Authors:** Sinziana Muraru, Carina Mihai, Muriel Elhai, Mike Becker, Suzana Jordan, Alexandru Garaiman, Cosimo Bruni, Liubov Petelytska, Anna-Maria Hoffmann-Vold, Oliver Distler, Rucsandra Dobrota

**Affiliations:** Department of Rheumatology, University Hospital Zurich, University of Zurich, Zurich, Switzerland; Department of Rheumatology, University Hospital Zurich, University of Zurich, Zurich, Switzerland; Department of Rheumatology, University Hospital Zurich, University of Zurich, Zurich, Switzerland; Department of Rheumatology, University Hospital Zurich, University of Zurich, Zurich, Switzerland; Department of Rheumatology, University Hospital Zurich, University of Zurich, Zurich, Switzerland; Department of Rheumatology, University Hospital Zurich, University of Zurich, Zurich, Switzerland; Department of Rheumatology, University Hospital Zurich, University of Zurich, Zurich, Switzerland; Department of Rheumatology, University Hospital Zurich, University of Zurich, Zurich, Switzerland; Department of Rheumatology, University Hospital Zurich, University of Zurich, Zurich, Switzerland; Department of Rheumatology, Oslo University Hospital, Oslo, Norway; Department of Rheumatology, University Hospital Zurich, University of Zurich, Zurich, Switzerland; Department of Rheumatology, University Hospital Zurich, University of Zurich, Zurich, Switzerland

**Keywords:** very early systemic sclerosis, arthritis, joint involvement

## Abstract

**Objective:**

Arthritis is associated with a worse prognosis in established SSc. However, knowledge about its relevance in very early SSc (veSSc) is scarce. We aimed to assess the prevalence and phenotype of arthritis, as well as its prognostic impact, in patients with veSSc.

**Methods:**

We analysed patients with veSSc, defined as presence of Raynaud’s phenomenon (RP) and/or at least one of: puffy fingers, antinuclear antibodies (ANA), abnormal capillaroscopy, not fulfilling the ACR/EULAR classification criteria for SSc at baseline. We investigated associations between arthritis and clinical parameters, followed by a longitudinal analysis to investigate arthritis as a potential predictor of progression towards established SSc.

**Results:**

We included 159 patients, of whom 108 had at least one follow-up visit. SSc-related arthritis occurred in 22/159 (13.8%) patients at baseline. Arthritis was mostly seronegative, symmetrical, oligo- or polyarticular, non-erosive, and rarely associated with elevation of inflammatory markers. More than half of the patients needed treatment with DMARDs. Anti-centromere antibodies (ACA) were negatively associated with arthritis (odds ratio 0.707, 95% CI 0.513–0.973, *P* = 0.033). Overall, 43/108 (39.8%) patients with follow-up progressed to established SSc during the observation time. Arthritis was not a significant predictor for progression to established SSc in a multivariable Cox regression.

**Conclusion:**

In this first comprehensive analysis, we found a similar prevalence of arthritis in veSSc as seen in established SSc. Moreover, the use of DMARDs indirectly suggests a relevant disease burden.

## Graphical Abstract

**Figure:**
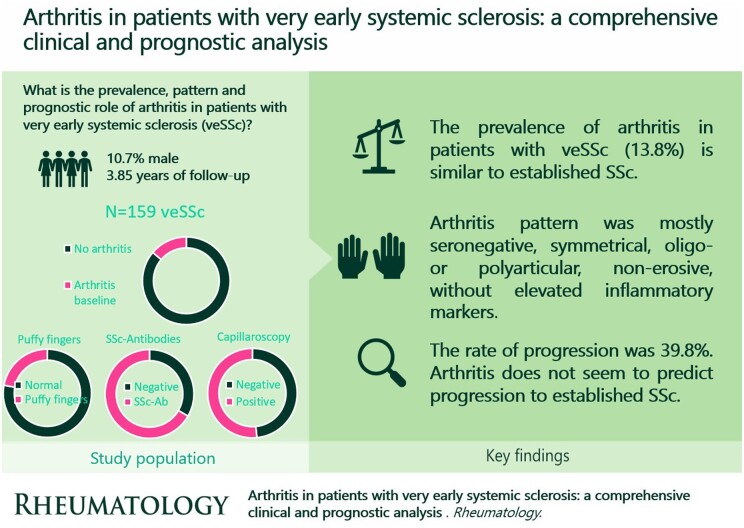

Rheumatology key messages

- The prevalence of arthritis in patients with very early SSc (veSSc) is similar to established SSc.
- Arthritis in veSSc is mostly seronegative, symmetrical, oligo- or polyarticular, non-erosive, and without elevated inflammatory markers.
- More than 50% of patients suffering from veSSc and arthritis need treatment with DMARDs.

## Introduction

SSc is a chronic, incurable, autoimmune connective tissue disease (CTD), associated with a high morbidity and mortality.

During the last two decades, experts raised awareness about the importance of early diagnosis of organ manifestations in the monitoring, treatment and prevention of complications [1]. Patients with ‘very early’ SSc (veSSc), also called pre-scleroderma, have disease features of SSc, such as Raynaud’s phenomenon (RP), puffy fingers and antinuclear antibodies (ANA), but no definite disease [1], and do not fulfil the 2013 ACR/EULAR classification criteria [2, 3]. Patients with veSSc show a heterogeneous pattern of disease manifestations and only some of them progress to established SSc [4]. The simultaneous presence of puffy fingers and SSc-specific antibodies carries the highest risk of progression towards SSc [5].

Little is known about other disease manifestations in veSSc, especially articular involvement. Joint involvement and arthritis specifically pose a relevant burden to patients with established SSc [6]. A comprehensive cross-sectional analysis of the European Scleroderma Trials and Research (EUSTAR) database including 7286 cases revealed the presence of any articular involvement (defined as synovitis, tendon friction rubs and contractures) in 28% of patients with established SSc, whereas isolated synovitis was reported in 16% [7]. Of these, 39% developed synovitis in early disease stages (<5 years disease duration) [7]. Joint and tendon involvement predicts a negative outcome in established SSc. In a large EUSTAR study, joint synovitis and tendon friction rubs independently predicted overall disease progression, defined as new onset or worsening of organ involvement [8]. Subsequent data demonstrated a predictive role of synovitis in worsening of skin fibrosis [9] and progression of interstitial lung disease (ILD) at 1-year follow-up [10]. Moreover, joint pain and arthritis, along with skin fibrosis and vasculopathy, represent important drivers of disability through impairment of hand function, fine motor skills and grip force in SSc patients [11, 12].

Despite their relevance for both prognosis and disability in established SSc, there are no data on the prevalence, phenotype and prognostic value of joint involvement in patients with very early disease stages. Therefore, our aim was to assess the prevalence and clinical pattern of arthritis in patients with veSSc, its association with other disease features and its potential prognostic impact.

## Methods

We included patients with veSSc, defined as presence of RP and/or at least one of the following disease features: puffy fingers, positive ANA, or SSc-pattern on nailfold capillaroscopy, who did not fulfil the 2013 ACR/EULAR classification criteria for SSc at baseline [3].

All patients were part of the local longitudinal EUSTAR/Very Early Diagnosis of Systemic Sclerosis (VEDOSS) cohort and signed an informed consent prior to their enrolment into the database. We included all recorded visits from March 2004 up to November 2022, with standardized data collection at every visit [13]. Data collection was approved by the Cantonal Ethics Committee of Zurich (EUSTAR EK-839, VEDOSS KEK-ZH-2010-0158/5) and according to the Declaration of Helsinki.

We defined arthritis as presence of synovitis, as assessed in the clinical examination by the treating physician at the time of the visit. In order to characterize in depth the veSSc-associated arthritis, we retrieved additional relevant information from the electronic medical records of the patients (Supplementary Table S1, available at *Rheumatology* online). We excluded cases of arthritis unrelated to SSc (crystal deposition disease, activated OA or reactive arthritis) based on a case-by-case judgement taking into consideration the list of diagnoses, the localization and pattern of the arthritis, the laboratory results and any available radiographs. We did not exclude cases of arthritis in context of overlap syndromes with other CTDs. Overlap syndromes were defined as fulfilment of the diagnostic or classification criteria (as available) for both diseases, e.g. veSSc and RA. To assess inflammatory bone erosions, an experienced rheumatologist with expertise in X-ray scoring (C.M.) evaluated the X-rays from the nearest time point to the visit reporting the presence of arthritis.

Disease duration was calculated since the first occurrence of the RP (if present), or, in patients without RP, since the first documented sign or symptom suggestive of SSc. Other disease characteristics were recorded according to EUSTAR standards [14].

We retrospectively screened the electronic records of patients with arthritis for treatment prescribed by their attending physician. We incorporated the use of analgesics and NSAIDs, glucocorticoids, conventional and biological DMARDs into our descriptive analysis.

ANA were tested by immunofluorescence and positivity was defined as any value above the upper normal limit of the local laboratory.

### Statistical methods

We performed an observational cohort study on prospectively collected data. Firstly, in a cross-sectional analysis of all baseline visits, we investigated associations between arthritis and relevant clinical parameters chosen by expert opinion, using Fisher’s test and Mann–Whitney U tests. We assessed (im)balance between groups of patients with/without arthritis using the standardized mean difference (SMD), which has the advantage of not being influenced by sample size or measurement unit [15, 16]. We considered the following cut-off values: SMD <0.2 small, 0.2–0.5 moderate, >0.8 large difference [17]. Variables largely imbalanced were further assessed in association tests to examine statistically significant differences between groups. Additionally, we assessed for differences among treated and untreated arthritis patients using the Mann–Whitney U test and the χ^2^ test, and applying the Bonferroni correction for multiple comparisons.

We further performed a longitudinal analysis using Kaplan–Meier plots and multivariable Cox regression in order to investigate whether arthritis could be a predictor of progression towards established SSc, defined as fulfilment of ACR/EULAR criteria at any follow-up visit. The covariates (specific antibodies, puffy fingers and SSc pattern on capillaroscopy) were selected according to expert opinion and literature [5].

We performed the statistical tests in R (version 4.1.3) and SPSS (version 29.0).

## Results

### Cohort characteristics

Of 737 SSc patients included in the local database, 159 fulfilled the inclusion criteria for veSSc and 108/159 had at least one follow-up visit, with a median follow-up of 2.0 years. The median age was 48 years, with a median disease duration of 3.0 years, and 17 (10.7%) were males. No patient had skin fibrosis at baseline (modified Rodnan skin score = 0).

The groups with and without arthritis were well balanced in terms of age, follow-up duration and rate of progression to established disease (SMD <0.2). We observed a moderate difference in autoantibody positivity, as more patients in the group without arthritis had anti-centromere antibodies (ACA) and anti-Scl70 antibodies (Table 1).

**Table 1.** Baseline characteristics of the entire veSSc cohort and stratified according to the presence of arthritis

Variables^a^	Total, *N* = 159	No arthritis at baseline, *N* = 137	SSc-arthritis at baseline, *N* = 22	SMD
Age (years), median (Q1–Q3)	48.00 (35.00, 59.50)	48.00 (35.00, 59.00)	53.50 (36.50, 60.75)	0.149
Male sex	17/159 (10.7)	15/137 (10.9)	2/22 (9.1)	0.062
Disease duration^b^ (years), median (Q1–Q3)	3.08 (1.17, 9.54)	3.08 (1.17, 9.17)	2.75 (1.35, 13.02)	0.277
Baseline visit only	51/159 (32.1)	45/137 (32.8)	6/22 (27.3)	0.122
Follow-up duration overall (years), median (Q1–Q3)	2.01 (0.00, 5.66)	1.98 (0.00, 5.64)	2.97 (0.24, 7.11)	0.163
Follow-up duration in patients with available follow-up visits (years), median (Q1–Q3)	3.85 (1.98, 8.10)	3.17 (1.98, 8.03)	4.91 (2.00, 8.23)	0.134
Fulfilled 2013 ACR/EULAR classification criteria for SSc during follow-up	43/108 (39.8)	37/92 (40.2)	6/16 (37.5)	0.056
Raynaud’s phenomenon	142/159 (89.3)	125/137 (91.2)	17/22 (77.3)	0.391
Digital ulcers	5/133 (3.8)	5/118 (4.2)	0/15 (0.0)	0.297
Pitting scars	2/134 (1.5)	2/119 (1.7)	0/15 (0.0)	0.185
Puffy fingers	32/145 (22.1)	26/128 (20.3)	6/17 (35.3)	0.339
Telangiectasia	18/157 (11.5)	13/141 (9.2)	5/16 (31.2)	0.570
ACA	85/159 (53.5)	78/137 (56.9)	7/22 (31.8)	0.522
Anti-Scl70 antibodies	13/157 (8.3)	13/135 (9.6)	0/22 (0.0)	0.462
Anti-RNA Polymerase III antibodies	12/141 (8.5)	10/123 (8.1)	2/18 (11.1)	0.101
SSc-specific antibodies (any of the three above at least once)	106/159 (66.7)	97/137 (70.8)	9/22 (40.9)	0.631
Elevated CRP (>5 mg/l)	17/151 (11.3)	15/129 (11.6)	2/22 (9.1)	0.083
Elevated ESR (>25 mm/h)	16/142 (11.3)	14/124 (11.3)	2/18 (11.1)	0.006
SSc pattern on nailfold capillaroscopy	82/159 (51.6)	70/137 (51.1)	12/22 (54.5)	0.069
Pulmonary hypertension on echocardiography	3/31 (9.7)	2/23 (8.7)	1/8 (12.5)	0.124
ILD on HRCT	9/133 (6.8)	6/115 (5.2)	3/18 (16.7)	0.373

a Frequencies are reported as *n*/valid cases and valid percentage.

b Disease duration since the first occurrence of RP if present, otherwise since the first documented SSc manifestation. *N*: number; veSSc: very early SSc; SMD: standardized mean difference; *n*: number of cases; anti-Scl70 antibodies: anti-topoisomerase I antibodies; ILD: interstitial lung disease; HRCT: high-resolution CT.

As expected for very early disease stages, the clinical phenotype of this cohort was mild, with rare complications or organ involvement: 5 (3.8%) of the patients had digital ulcers, 2 (1.5%) pitting scars and 9 (6.8%) had signs of ILD on high-resolution CT at baseline (Table 1).

### Characterization of the phenotype of arthritis in very early SSc

In the whole veSSc cohort, 32/159 (20.1%) patients had at least one visit with evidence of arthritis. After reviewing the clinical records, we excluded 2/32 patients with arthritis that was most likely related to calcium pyrophosphate deposition disease. Among the remaining 30 SSc-related arthritis cases, there were 22 patients having SSc-related arthritis at baseline and four during follow-up visits not fulfilling the classification criteria. The remaining four cases developed arthritis after being classified as established SSc.

Table 2 shows the clinical characteristics of all patients with veSSc and arthritis, as well as laboratory parameters, relevant comorbidities and treatment.

**Table 2.** Characteristics of veSSc patients having SSc-related arthritis at any visit during follow-up (*N* = 26)

Parameter	*N*, % or median
General	
Age, median (years) (IQR, Q1–Q3)	55 (16.75, 44.25–61)
Sex, female	23/26 (88.46)
Follow-up time (years), median (IQR, Q1–Q3)	2.05 (5.20, 0.94–6.14)
Disease duration^a^ (years), median (IQR, Q1–Q3)	3.50 (12.50, 1.46–13.96)
Fulfilled criteria during follow-up	6/16 (37.50)
Current or ever smokers	12/26 (46.15)
SSc-related arthritis at baseline	22/26 (84.62)
SSc-related arthritis at later visits not fulfilling the 2013 ACR/EULAR classification criteria for SSc	4/26 (15.38)
Monoarticular	3/25 (12.00)
Oligoarticular (>1, <5 joints)	11/25 (44.00)
Polyarticular (≥5 joints)	11/25 (44.00)
Symmetrical	19/25 (76.00)
Tenosynovitis	5/25 (20.00)
Tendon friction rubs	1/25 (4.00)
Synovitis confirmed by US^b^	11/12 (91.67)
Erosive disease on X-ray	4/23 (17.39)
Laboratory parameters	
CRP elevation (>5 mg/l)	2/26 (7.69)
ESR elevation (>25 mm/h)	3/26 (11.54)
RF	6/26 (23.08)
ACPA	1/24 (4.17)
ANA	24/26 (92.31)
ANA titres, median (Q1–Q3)	1:2560 (1:1120-1:10240)
Isolated ANA	8/24 (33.33)
ACA	8/26 (30.77)
Anti-RNA Polymerase III antibodies	2/24 (8.33)
Relevant comorbidities	
Overlap seropositive RA	1/26 (3.85)
Overlap features with myositis, SLE	3/26 (11.54)
Degenerative disease of the hand joints^c^	12/24 (50.00)
CPPD	1/26 (3.85)
FM	3/26 (11.54)
Treatment	
Analgesics and/or NSAIDs	17/26 (65.38)
Glucocorticoids any dose	8/26 (30.77)
Conventional or biological DMARDs^d^ monotherapy	14/26 (53.85)
Conventional or biological DMARDs^d^ combination therapy	2/26 (7.69)

a Disease duration since the first occurrence of RP if present, otherwise since the first documented disease manifestation.

b Performed according to the treating physician.

c Trapeziometacarpal OA, scaphotrapeziotrapezoid joint OA, OA of the fingers.

d Conventional DMARDs prescribed were: HCQ, MTX, SSZ, LEF, MMF; biologicals considered were: tocilizumab, rituximab. *N*: number; IQR: interquartile range; veSSc: very early SSc; CPPD: calcium pyrophosphate deposition disease.

The pattern of joint involvement was in most cases symmetrical, oligo- or polyarticular, affecting mostly wrist- and finger joints, and only rarely larger joints like the elbow (1/26), knee (1/26) and ankle (2/26). Twenty percent of the patients also had tenosynovitis. ultrasound exams confirmed the clinical diagnosis of arthritis in almost all cases where it was performed (11/12), but were available only in about half of the patients (12/26), since it was performed as judged by the treating physician. Erosive disease on X-ray was found in a minority of patients 4/23 (17.39%), most of whom (3/4), however, showed radiological progression at follow-up (Supplementary Material, available at *Rheumatology* online).

Surprisingly, only a few patients had elevated inflammatory markers (2/26 CRP, 3/26 ESR) and 23% had a positive RF.

Patients with arthritis and veSSc had positive ANA in about 80% of the cases. Interestingly, patients with arthritis showed less frequently SSc-specific antibodies (ACA, anti-Scl-70 and anti-RNA Polymerase III) than patients without arthritis (SMD 0.631). This is driven by the lower prevalence of ACA in patients with arthritis (SMD 0.522, Table 1) and the lack of anti-Scl-70 antibodies in the arthritis group. However, about two-thirds of the patients did not have any of the three antibodies mentioned above. Among these, we found four cases positive for PM-Scl antibodies and two cases having anti-fibrillarin and anti-NOR90 antibodies, respectively (Supplementary Table S2, available at *Rheumatology* online). Another one-third of the cases had isolated ANAs (32%).

The DAS28-ESR (DAS-CRP respectively) severity score was available only in 6/26 patients at visits having arthritis and ranged from 2.16 (1.75) up to 7.53 (5.92).

More than half of the patients needed treatment with NSAIDs, whereas only about one-third received glucocorticoids. Moreover, in 16/26 patients, a more intensive arthritis treatment with either conventional or biological DMARDs was necessary. The conventional DMARDs prescribed were HCQ, MTX, SSZ and LEF, whereas some patients were under tocilizumab or rituximab as biologic DMARDs. According to the electronic records, the main indication for the latter was the presence of arthritis as judged by the attending physician. Among these cases, two patients additionally received MMF because of myositis. The treated group contained more patients with erosive disease, elevated RF and specific SSc antibodies (Supplementary Table S3, available at *Rheumatology* online). However, these differences were not statistically significant after applying the Bonferroni correction (Supplementary Table S4, available at *Rheumatology* online).

There was one case of overlap with seropositive RA also having gout, and three other overlap syndromes with other CTDs (myositis or SLE). Despite the high prevalence of OA as a comorbidity in over 50% of the patients, the pattern of joint involvement and laboratory findings of those developing franc arthritis enabled their classification under an inflammatory aetiology. The prevalence of hand OA in patients with arthritis was slightly lower compared with the patients without arthritis (58.5%), for whom clinical and/or radiographic data were available (Supplementary Material, available at *Rheumatology* online).

### Cross-sectional associations between clinical parameters and arthritis in very early SSc at baseline

We further investigated for possible associations between the presence of arthritis at baseline and relevant clinical and laboratory parameters (Table 3).

**Table 3.** Cross-sectional associations between veSSc arthritis and relevant parameters at the baseline visit (*N* = 22)

		Arthritis		*P*-value^a^
		Yes	No	
ACA	Positive	7	78	**0.038**
	Negative	15	59	
Anti-Scl70 Ab	Positive	0	13	0.217
	Negative	22	122	
Anti-RNA Polymerase III Ab	Positive	2	10	0.652
	Negative	16	113	
Puffy fingers	Present	6	26	0.210
	Absent	11	102	
CRP level >5 mg/l	Present	2	15	>0.999
	Absent	20	114	
ESR level >25 mm/h	Present	2	14	>0.999
	Absent	16	110	
Disease duration^b^	<3 years	9	58	>0.999
	>3 years	9	59	

a Fisher’s exact test with the two-sided *P-*value reported. **Bold** ***P*** values are statistically significant.

b Calculated since the first occurrence of RP, if present, otherwise since the first documented SSc manifestation. veSSc: very early SSc; Ab: antibodies; anti-Scl70: anti-topoisomerase I.

Patients with arthritis had significantly less frequent ACA (*P* = 0.038, Table 3). This association was confirmed in a binary logistic regression with the outcome presence of arthritis: we obtained an odds ratio for ACA of 0.707 (95% CI 0.513–0.973, *P* = 0.033), suggesting a potential protective role in the development of arthritis. Arthritis was associated with neither the other SSc-specific antibodies (anti-Scl70 and anti-RNA-Polymerase III) nor with inflammatory markers, presence of puffy fingers or disease duration (Table 3).

### Longitudinal and prognostic analysis of arthritis in very early SSc

**Figure 1.**
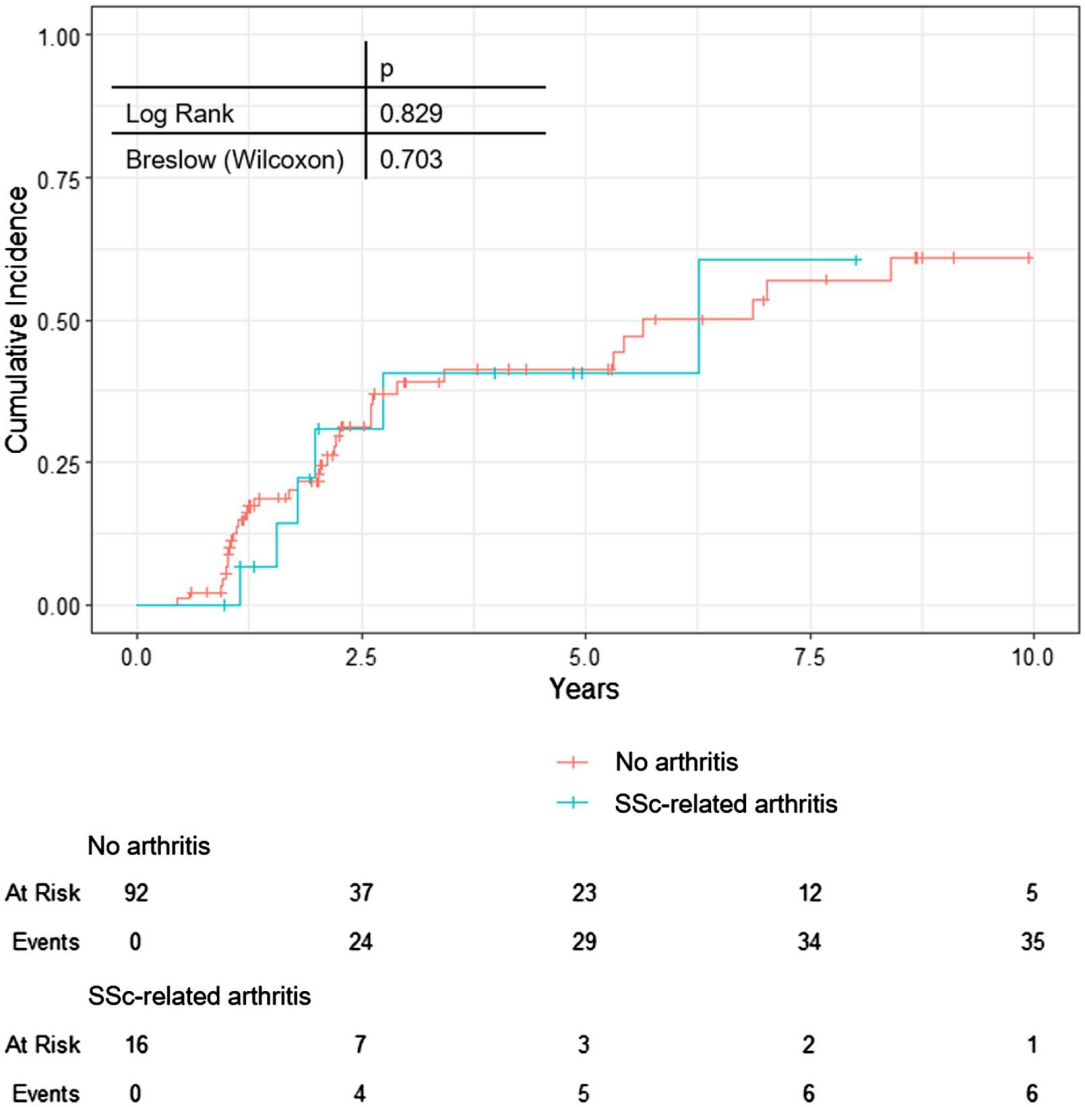
Cumulative incidence of progression to SSc stratified by very early SSc-related arthritis (Kaplan–Meier, 1 – cumulative survival)

More than one-third of the patients with recorded follow-up (43/108, 39.8%) progressed to established disease during the observation time (defined as fulfilling of the 2013 ACR/EULAR classification criteria). We further investigated whether patients with arthritis had a higher risk of progression towards established SSc, using Kaplan–Meier and Cox regression. There was no statistically significant difference in fulfilling the classification criteria at follow-up between patients with and without arthritis (Fig. 1).

Furthermore, arthritis was not a significant independent predictor for progression to established SSc in a multivariable Cox regression model with the outcome fulfilment of the classification criteria and adjusted for known predictors (specific antibodies, puffy fingers and SSc pattern on capillaroscopy) [5]. In this model, only the SSc-specific antibodies (*P* = 0.024, hazard ratio 1.386, 95% CI 1.043–1.840) reached significance (Supplementary Table S5A and B, available at *Rheumatology* online).

## Discussion

In this first comprehensive characterization of arthritis in a large cohort of veSSc patients, we report a relevant point prevalence (22/159 at baseline, 13.8%; 26/159 including follow-up visits without fulfilling criteria, 16.4%), similar to the prevalence reported in established SSc (16–18%) [7, 8]. Arthritis in veSSc was mostly seronegative and non-erosive, having a symmetrical, oligo- or polyarticular involvement, with rare elevation of inflammatory markers [overall, 3/22 (13.64%) had at least one elevated marker]. This is different compared with findings in established SSc, where elevated acute-phase reactants were reported in 24% [8], and up to 45% of the cases with arthritis [7].

Despite the apparently mild profile, >50% of the patients needed treatment with conventional or biological DMARDs, as monotherapy or in combination. These data highlight arthritis as a relevant burden in patients with veSSc, which should be thoroughly assessed in clinical practice.

Patients with veSSc and ACA had significantly less arthritis. A large EUSTAR cohort study showed similar results in established SSc, but the result was not significant in the adjusted analyses [7]. ACA tend to occur mostly in patients with the limited cutaneous disease subset [18], whereas arthritis is more prevalent in the diffuse cutaneous disease subset [7]. A potential protective role of ACA should be confirmed in future longitudinal studies. Interestingly, none of the patients with Scl70 antibodies showed arthritis in our cohort. Since ours is the first report focusing on arthritis in veSSc, no data are available for comparison with similar veSSc cohorts. In cohorts of established SSc, the prevalence of anti-Scl70 antibodies in SSc patients with arthritis was higher, up to half of the included cases: 497/1191 (42%) [5] and 120/234 (51%) [8].

There was no significant difference in disease duration between patients with and without arthritis. This is similar to arthritis of established SSc, which may occur in all disease stages [7].

Strengths of our study include the longitudinal follow-up of the cohort with standardized in depth assessments and a relatively high number of veSSc patients. Furthermore, we comprehensively characterized veSSc arthritis, integrating registry data and clinical records, as well as an additional expert assessment of the radiographic images for inflammatory erosive disease. These data improve the understanding of the clinical, immunological and radiological pattern as well as of the burden of arthritis in veSSc. Our study can thus increase awareness of arthritis in veSSc and inform the clinician to optimize the management of these patients.

There are several limitations to our study. First, the clinical assessment of synovitis in the presence of puffy fingers might be challenging, even for experienced physicians. However, although the group with arthritis had a higher prevalence of puffy fingers compared with the overall cohort, only 6 patients had both arthritis and puffy fingers, whereas 11 patients with synovitis did not have puffy fingers. There was also no statistically significant association between arthritis and puffy fingers in this cohort. As such, we do not consider this a major confounder. Second, episodes of synovitis from in-between the yearly visits were not documented. Thus, the point prevalence of arthritis might be underreported, as data about arthritis flares occurring at different time points and/or treated by other physicians were not available. In order to overcome the potential limitation of analysing arthritis cases of other causes, especially by associated OA, we manually verified the electronic records of the included patients and updated the data accordingly.

In this mild cohort, arthritis did not predict progression to established SSc, defined using the SSc classification criteria as a surrogate measure [5]. However, the clinical diagnosis of SSc in practice does not depend on fulfilment of these criteria. Because specific antibodies have a high contribution to the total classification score (3/9 points [3]), we checked whether we might have enriched our cohort with antibody-negative cases. This was not the case, since our overall veSSc study cohort had an even higher percentage of specific antibodies (ACA, anti-RNA Polymerase III and anti-Scl70 antibodies) (66.9%) than reported in the literature for veSSc (53–56.5%) [5, 19] and closer to the prevalence in established SSc (71.5%) [14]. Our data are also in line with other veSSc cohorts regarding rate of progression, overall follow-up time, age and distribution of specific antibodies. The prevalence of the anti-Scl70 antibodies in our cohort (8.3%) is similar [5] or only slightly lower than in international veSSc cohorts (11.3–14.5%) [19, 20] (Supplementary Table S6, available at *Rheumatology* online), whereas the prevalence of anti-RNA III Polymerase antibodies was higher in our population (8.5%) than in multiple cohorts of veSSc (1.7–3.3%) [5, 19, 20] (Supplementary Table S6, available at *Rheumatology* online).

Significant disease progression, however, was rare. During the observation time, only one patient from the whole cohort and none with arthritis developed diffuse skin fibrosis and 16/108 (among whom only 4 had arthritis) developed ILD. This is in line with a Brasilian population of veSSc with the same inclusion criteria, among which there was no case of skin fibrosis during the entire follow-up [20]. Therefore, despite the relevant sample size, our cohort includes mostly mild veSSc cases with progression towards lcSSc or SSc sine scleroderma within the available observation time. There is a lower likelihood of including patients with anti-Scl70 or anti-RNA Polymerase III, who face a higher risk of progression, into a veSSc cohort using the current definition based on non-fulfilment of the ACR/EULAR classification criteria [5]. This is caused by the short time window of opportunity for the inclusion, before additional signs and symptoms classifying these patients as established SSc occur. Mild veSSc cohorts are thus different from established early SSc cohorts.

We need further studies on larger cohorts and longer follow-up in order to understand whether the negative prognostic role of arthritis for the development of fibrosis described in established SSc [8–10] also applies to veSSc. Further investigation of the prognostic role of arthritis in specific subgroups of patients with veSSc (e.g. with anti-Scl70 antibodies or inflammation) and for development of specific organ complications would be of particular interest.

Moreover, knowing the progressive nature of the disease with cumulative damage of the musculoskeletal structures, an early treatment of the inflammatory component may improve at least partially the hand function, one of the most important domains limiting the wellbeing of SSc patients [21, 22].

In conclusion, our study raises awareness about the relevance of arthritis in patients with veSSc, supporting screening for arthritis alongside other major organ involvement.

## Supplementary Material

keae247_Supplementary_Data

## Data Availability

Anonymized data can be made available from RD at the Department of Rheumatology, University Hospital Zurich, University of Zurich, Switzerland upon reasonable request.
