# Supplementary material for: Arthritis in patients with very early systemic sclerosis: a comprehensive clinical and prognostic analysis
Source: Rheumatology (Oxford). 2024 May 9;64(3):1243–50. doi: 10.1093/rheumatology/keae247 (PMC11879340; doi:10.1093/rheumatology/keae247)
Supplement: keae247_Supplementary_Data [file keae247_supplementary_data.docx]

**Supplementary methods**

**Variable Types**

**Supplementary Table S1.** Additional variables retrieved and summarized from the clinical electronic records of patients with arthritis and veSSc.

| **Additional Variables** | **Values** | **Comments** |
| --- | --- | --- |
| Visit Date | DD/MM/YYYY |  |
| First diagnosis of arthritis mentioned | DD/MM/YYYY |  |
| Synovitis at baseline | Yes/No |  |
| Synovitis at later visits without fulfilling classification criteria | Yes/No |  |
| Recurrent arthritis | Yes/No | Arthritis described at more than 2 visits |
| Monoarticular involvement | Yes/No |  |
| Oligoarticular involvement | Yes/No |  |
| Polyarticular involvement | Yes/No |  |
| Symmetrical involvement | Yes/No |  |
| Exact joints with arthritis | Text |  |
| Swollen joint count | Number |  |
| Tender joint count | Number |  |
| Tenosynovitis | Yes/No |  |
| Tendon friction rubs | Yes/No |  |
| Morning stiffness | Yes/No |  |
| Date of X-Ray of the involved joints | DD/MM/YYYY |  |
| Signs of degenerative osteoarthritis of the hand | Yes/No |  |
| Erosions not attributed to degenerative changes | Yes/No |  |
| Presence of synovitis on ultrasound | Yes/No |  |
| Ultrasound date | DD/MM/YYYY |  |
| Rheumatoid factor | Yes/No |  |
| Anti-CCP antibodies | Yes/No |  |
| CRP elevation (>5mg/l) | Yes/No |  |
| ESR elevation (>25mm/h) | Yes/No |  |
| DAS28-CRP | Number |  |
| DAS28-ESR | Number |  |
| Cigarette smoking ever | Yes/No |  |
| Current cigarette smoker | Yes/No |  |
| Cigarette pack years | Number |  |
| CPPD | Yes/No |  |
| Gout | Yes/No |  |
| Fibromyalgia | Yes/No |  |
| Overlap syndromes | Text | Overlap with rheumatoid arthritis or other connective tissue diseases |
| Analgesia | Yes/No | Both NSAIDs and other analgetic drugs (paracetamol, opioids) |
| NSAIDs or Cox-2 inhibitors | Yes/No |  |
| Systemic glucocorticoids | Yes/No |  |
| Intraarticular glucocorticoids | Yes/No |  |
| Conventional and biologic DMARDs | Yes/No |  |
| Comment which DMARD/B | Text |  |
| Treatment start date | DD/MM/YYYY |  |
| Treatment end date | DD/MM/YYYY or text if ongoing |  |
| Abbreviations: DD/MM/YYYY Day/Month/Year; Anti-CCP anti-citrullinated protein antibodies; CRP C-reactive protein; ESR erythrocyte sedimentation rate; DAS-28 Disease Activity Score in 28 joints; CPPD calcium pyrophosphate deposition disease; NSAIDs non-steroidal anti-inflammatory drug; Cox-2 cyclooxygenase-2; DMARDs disease modifying anti-rheumatic drugs. | | |

**Supplementary results**

**Supplementary Table S2.** Detailed profile of the antibodies in patients with veSSc related arthritis.

| **Antibodies** | **N** | **Additional positive serology (if available)** |
| --- | --- | --- |
| ANA-negative | 2/26 |  |
| Anti-centromere Ab | 8/26 | Anti-Mi-2 Ab (1/8), anti-SSA (1/8) |
| Anti-Scl-70 Ab | 0/26 |  |
| Anti-RNA-Pol-III Ab | 2/24 | Anti-Pm/Scl and anti-NOR-90 Ab (1/2) |
| Anti-fibrillarin Ab | 2/19 | Anti-Ku Ab (1/2) |
| Anti-PM/Scl Ab | 4/23 | Anti-RNA-Pol-III and anti-NOR-90 Ab (1/4) |
| Anti-Ku Ab | 1/19 | Anti-fibrillarin Ab (1/1) |
| Anti-NOR-90 Ab | 2/13 | Anti-Pm/Scl and anti-RNA-Pol-III Ab (1/2) |
| Anti-SSA | 2/25 | Anti-Centromere (1/2) |
| N number of cases of available; ANA antinuclear antibodies; Ab antibodies; Anti-Scl 70 Ab anti-topoisomerase I antibodies; Anti RNA-Pol-III Ab anti ribonucleic acid polymerase III antibodies; Anti-Pm/Scl Ab anti-polymyositis-scleroderma antibodies; Anti-NOR-90 Ab anti nucleolus-organizing regions 90 kDa antibodies; Anti-SSA anti–Sjögren's-syndrome-related antigen A autoantibodies | | |

The group of patients receiving DMARDs was numerically different compared to the group without treatment in terms of having a shorter median disease duration, including more smokers, having more often tenosynovitis and tendon friction rubs, as well as more erosions on X-Ray not attributed to degenerative osteoarthritis according to the specialized rheumatologist (CM). Moreover, the serologic profile showed a lower prevalence of specific antibodies included in the classification criteria in the untreated group (Table S2). However, the differences were not statistically significant in this small sample, after applying the Bonferroni correction for multiple comparisons (Table S3).

**Supplementary Table S3.** Profile of the SSc-arthritis patients with and without DMARD treatment (main indication being the arthritis).

| Variables | Overall  (N=26) | No DMARDs (N=11) | DMARDs^a^ (N=15) | SMD |
| --- | --- | --- | --- | --- |
| **General** | N (%) or median [Q1-Q3] | | | |
| Age, median [Q1-Q3] (years) | 55 [44.25-61] | 46.00 [37.50, 56.50] | 59.00 [52.00, 65.50] | 0.639 |
| Sex, Male | 3 (11.5) | 2 (18.2) | 1 (6.7) | 0.355 |
| Follow-up time, median [Q1-Q3] (years) | 2.05 [0.94, 6.14] | 2.07 [0.65, 7.80] | 1.92 [0.95, 4.91] | 0.345 |
| Disease duration^b^, median [Q1-Q3] (years) | 3.25 [1.33, 26.17] | 5.92 [2.50, 23.29] | 1.42 [0.67, 15.04] | 0.238 |
| Fulfilled criteria during the follow-up | 6 (23.1) | 2 (18.2) | 4 (26.7) | 0.204 |
| Current or ever smokers | 12 (50.0) | 4 (36.4) | 8 (61.5) | 0.520 |
| SSc-related synovitis at baseline | 22 (84.6) | 9 (81.8) | 13 (86.7) | 0.133 |
| SSc-related synovitis at later visits not fulfilling criteria | 4 (17.4) | 1 (12.5) | 3 (20.0) | 0.204 |
| Monoarticular | 3 (12.0) | 1 (10.0) | 2 (13.3) | 0.104 |
| Oligoarticular (>1, <5 joints) | 11 (44.0) | 5 (50.0) | 6 (40.0) | 0.202 |
| Polyarticular (>=5 joints) | 11 (44.0) | 4 (40.0) | 7 (46.7) | 0.135 |
| Symmetrical | 19 (76.0) | 7 (70.0) | 12 (80.0) | 0.232 |
| Tenosynovitis | 5 (20.0) | 1 (9.1) | 4 (28.6) | 0.514 |
| Tendon friction rubs | 1 (3.8) | 1 (9.1) | 0 (0.0) | 0.447 |
| Erosive disease on X-Ray | 4 (17.4) | 0 (0.0) | 4 (28.6) | 0.894 |
| **Laboratory parameters** |  | | | |
| CRP Elevation | 2 (7.7) | 1 (9.1) | 1 (6.7) | 0.090 |
| ESR Elevation | 3 (11.5) | 1 (9.1) | 2 (13.3) | 0.135 |
| Rheumatoid factor | 6 (23.1) | 0 (0.0) | 6 (40.0) | 1.155 |
| ACPA | 1 (4.2) | 0 (0.0) | 1 (7.7) | 0.408 |
| ANA | 24 (92.3) | 10 (90.9) | 14 (93.3) | 0.090 |
| Antibodies anti-Centromere | 8 (30.8) | 1 (9.1) | 7 (46.7) | 0.923 |
| Specific antibodies (anti-Centromere, anti-RNA Polymerase III)^c^ | 10 (38.5) | 1 (9.1) | 9 (60.0) | 1.268 |
| DMARDs disease modifying anti-rheumatic drugs; N Number; SMD standardized mean difference; SSc systemic sclerosis; CRP C-reactive protein; ESR erythrocyte sedimentation rate; ACPA anti-citrullinated protein antibodies; ANA antinuclear antibodies; anti-RNA Polymerase III anti-ribonucleic acid Polymerase III  ^a^with the main indication being arthritis according to patients records; conventional or biological DMARDs in mono- or combination therapy;  ^b^since Raynaud or onset of first non-Raynaud manifestation (if no Raynaud at baseline)  ^c^there was no patient with anti-Scl-70 (anti-topoisomerase I) antibodies in this arthritis cohort | | | | |

**Supplementary Table S4.** Differences between the treated and untreated groups of patients with veSSc-related arthritis are not statistically significant.

| Variables | No DMARDs (N=11) | DMARDs (N=15) | SMD | p (MWU or Chi-square) |
| --- | --- | --- | --- | --- |
|  | N (%) or median [Q1-Q3] | | | |
| Age, median [Q1-Q3] (years) | 46.00 [37.50, 56.50] | 59.00 [52.00, 65.50] | 0.639 | 0.087 |
| Current or ever smokers | 4 (36.4) | 8 (61.5) | 0.520 | 0.219 |
| Associated tenosynovitis | 1 (9.1) | 4 (28.6) | 0.514 | 0.227 |
| Erosive disease on X-Ray | 0 (0.0) | 4 (28.6) | 0.894 | 0.078 |
| Rheumatoid factor | 0 (0.0) | 6 (40.0) | 1.155 | 0.017 ^a^ |
| Antibodies anti-Centromere | 1 (9.1) | 7 (46.7) | 0.923 | 0.079 |
| Specific antibodies (anti-Centromere, anti-RNA Polymerase III)^b^ | 1 (9.1) | 9 (60.0) | 1.268 | 0.019 ^a^ |
| DMARDs disease modifying anti-rheumatic drugs; N Number; SMD standardized mean difference; MWU Mann-Whitney U test; anti-RNA Polymerase III anti-ribonucleic acid Polymerase III  ^a^not significant after applying the Bonferroni correction with the new significance level at p=0.007  ^b^there was no patient with anti-Scl-70 (anti-topoisomerase I) antibodies in this arthritis cohort | | | | |

**Supplementary Table S5A.** Cox regression model with the outcome fulfillment of the ACR/EULAR classification criteria and adjusted for arthritis and known predictors of progression to SSc (presence of specific antibodies, SSc-pattern on nailfold capillaroscopy and puffy fingers).

|  | **Hazard ratio** | **95% CI** | **P-Value** |
| --- | --- | --- | --- |
| Arthritis | 1.347 | 0.505 to 3.596 | 0.552 |
| Specific antibodies | 1.386 | 1.043 to 1.840 | 0.024 |
| SSc pattern on nailfold capillaroscopy | 0.961 | 0.704 to 1.447 | 0.875 |
| Puffy fingers | 1.451 | 0.983 to 2.141 | 0.061 |
| CI confidence interval; SSc systemic sclerosis | | | |

Table S5B. Cox regression model with the outcome fulfillment of the ACR/EULAR classification criteria and adjusted for arthritis and known predictors of progression to SSc (presence of ANA, SSc-pattern on nailfold capillaroscopy and puffy fingers).

|  | **Hazard ratio** | **95% CI** | **P-Value** |
| --- | --- | --- | --- |
| Arthritis | 0.992 | 0.376 to 2.620 | 0.987 |
| ANA | 0.941 | 0.202 to 4.372 | 0.938 |
| SSc-pattern on nailfold capillaroscopy | 0.876 | 0.620 to 1.239 | 0.455 |
| Puffy fingers | 1.288 | 0.888 to 1.868 | 0.183 |
| CI confidence interval; ANA anti-nuclear antibodies; SSc systemic sclerosis | | | |

Supplementary Table S6. Prevalence of SSc-specific antibodies in very early systemic sclerosis reported in the literature and in our study.

| **Antibodies**  **N/available (%)** | **Anti-Centromere** | **Anti-Scl-70** | **Anti-RNA-Polymerase III** |
| --- | --- | --- | --- |
| Minier et all, 2013 [2]  (n= 318, 33 EUSTAR centers) | 135/310(43.5) | 35/310(11.3) | 2/116(1.7) |
| Siqueira et all, 2022 [3]  (n= 152, Brazil) | 44/NA (62) | 12/NA (14.5) | 0/39 (0)^a^ |
| Bellando-Randone et all, 2021 [1]  (n= 553, 42 EUSTAR centers) | 164/519 (31.6) | 39/525 (7.4) | 6/180 (3.3) |
| Our study  (n=159, Zurich) | 85/159 (53.5) | 13/157 (8.3) | 12/141 (8.5) |
| ^a^not tested at baseline, 39 patients with retrospective testing had negative status | | | |

Radiologic progression

Radiographs were repeated in 11 patients, after a median time of 5.5 years. Among them, 6 were stable, 1 had isolated progression secondary to arthritis, 2 had isolated progression of the degenerative changes and 2 had both progression of the inflammatory and degenerative changes. Interestingly, 3/4 patients with erosive inflammatory disease at the initial radiograph had progression of the changes secondary to the inflammation, either isolated or combined with a progression of degenerative changes.

The prevalence of hand osteoarthritis in our cohort

Incorporating a cumulative diagnosis derived from both radiographic and clinical data, the overall prevalence of degenerative changes of the hand was 46/81 (56.8%).

The prevalence in patients with veSSc and arthritis (N=26) was 12/24 (50.0%). If we include the additional 4 patients who developed arthritis at visits fulfilling the classification criteria, this number would increase to 15/28 (53.6%).

Thus, the prevalence of hand osteoarthritis in arthritis patients is slightly lower than the one observed in patients without SSc-related arthritis (31/53, 58.5%) in our cohort.
